# Hypothyroidism among Children with Nephrotic Syndrome Admitted to a Tertiary Care Centre

**DOI:** 10.31729/jnma.8455

**Published:** 2024-02-29

**Authors:** Subhana Thapa Karki, Najala Khatun, Ram Hari Chapagain, Nisha Jyoti Shrestha, Sumit Agrawal

**Affiliations:** 1 Department of Pediatric Medicine, National Academy of Medical Sciences, Mahaboudha, Kathmandu, Nepal; 2 Department of Pediatric Medicine, Kanti Children's Hospital, Maharajgunj, Kathmandu, Nepal

**Keywords:** *children*, *hypothyroidism*, *nephrotic syndrome*, *prevalence*

## Abstract

**Introduction:**

Nephrotic syndrome is a glomerular disease characterized by massive urinary protein loss occurring in children. Proteinuria also leads to loss of thyroid binding globulin affecting thyroid function. This study aimed to find out the prevalence of hypothyroidism among children with nephrotic syndrome admitted to a tertiary care centre.

**Methods:**

A descriptive cross-sectional study was conducted among children with nephrotic syndrome admitted to a tertiary care centre from 06 July 2020 to 06 June 2021 after obtaining ethical approval from the Ethical Review Committee. They were tested for free T3, free T4 and TSH. A convenience sampling method was used. The point estimate was calculated at a 90% Confidence Interval.

**Results:**

Among 69 children with nephrotic syndrome, the prevalence of hypothyroidism was 49 (71.01%) (62.03-80.00, 90% Confidence Interval).

**Conclusions:**

The prevalence of hypothyroidism among children with nephrotic syndrome was higher than other studies done in similar settings.

## INTRODUCTION

Proteinuria in nephrotic syndrome leads to loss of albumin and thyroxine-binding globulin (TBG) and can affect thyroid function. In nephrotic syndrome, renal tubules are damaged and reduce the reabsorption of low-molecular-weight proteins, including free thyroid hormones. Other factors besides heavy proteinuria may be involved in changes in thyroid function.^1^

Defect in the functioning of either organ is expected to have consequences on both systems.^2^ Many researchers have found the hypothyroid state in nephrotic syndrome and normalization of thyroid function during remission of nephrotic syndrome.^2^ The timely recognition and treatment of the hypothyroid state in nephrotic syndrome can prevent further consequences of hypothyroidism and nephrotic syndrome may also go into remission in a shorter time than usual.^3^

This study aimed to find out the prevalence of hypothyroidism among children with nephrotic syndrome admitted to a tertiary care centre.

## METHODS

This was a descriptive cross-sectional study conducted among children with nephrotic syndrome admitted to the Endocrinology and Nephrology unit of Kanti Children's Hospital, Maharajgunj, Kathmandu, Nepal. The data was collected from 06 July 2020 to 06 June 2021 after getting approval from the Ethical Review Committee (Registration number: 31/2020). All children of age below 14 years admitted with a diagnosis of nephrotic syndrome were included in the study. Those children whose parents did not give consent were excluded from the study. A convenience sampling method was used. The sample size was calculated by using the following formula:


$$\begin{array}{ll} \text{n} & ={\text{Z}}^{2}\times \frac{\text{p}\times \text{q}}{{\text{e}}^{\text{2}}}\\ & =1.96^{2}\times \frac{0.452\times 0.548}{0.1^{2}}\\ & =67 \end{array}$$

Where,

n = Sample size
Z = 1.96 at 95% confidence interval
p = prevalence taken from the previous study, 41.16^1^
q = i-p
e = margin of error, 10%

The calculated sample size was 67. However, we included 69 children in our study.

Children with nephrotic syndrome were classified as children with initial episodes, frequent relapse, infrequent relapse, steroid-dependent, steroid-resistant and remission cases.^4^ Apart from the routine tests that were done for all these children, free T3, free T4 and TSH levels at admission and after remission were sent. Subclinical hypothyroidism was defined as elevated TSH with normal free T4 levels and hypothyroidism was defined as elevated TSH and low free T4 levels.^5^

Data was entered and analysed using IBM SPSS Statistics version 25.0. The point estimate was calculated at a 90% CI.

## RESULTS

Among 69 children with nephrotic children, the prevalence of hypothyroidism was 49 (71.01%) (62.03-80.00, 90% CI). Among them, subclinical hypothyroidism was seen in 27 (55.10%) and overt hypothyroidism in 22 (44.90%) children. The mean age at the time of diagnosis was 7.8±4.6 years. A total of 34 (69.39%) were male (Table 1). The mean TSH at the time of diagnosis was 8.39±6.75 mlU/l and after remission was 4.03±2.93 mlU/l.

**Table 1:** Age and sex-wise distribution among hypothyroid children with nephrotic syndrome (n= 49).

Thyroid function profile	<2 years		**2 to 6 years**		**6 to 10 years**		**>10 years**	
	Male n(%)	Female n(%)	Male n(%)	Female n(%)	Male n(%)	Female n(%)	Male n(%)	Female n(%)
Subclinical hypothyroidism	2 (4.08)	1 (2.04)	9 (18.37)	3 (6.12)	4 (8.16)	5 (10.20)	2 (4.08)	1 (2.04)
Hypothyroidism	1 (2.04)	0	8 (16.33)	2 (4.08)	6 (12.24)	2 (4.08)	2 (4.08)	1 (2.04)

On the first episode of nephrotic syndrome, subclinical hypothyroidism was seen in 15 (30.61%) and overt hypothyroidism in 10 (20.41%) (Table 2).

**Table 2:** Hypothyroidism in different types of nephrotic syndrome at the time of diagnosis (n = 49).

Thyroid hormone profile	**Type of nephrotic syndrome**				
	First episode n(%)	Frequent relapse n(%)	Infrequent relapse n(%)	Steroid dependent n(%)	Steroid resistant n(%)
Subclinical hypothyroidism	15 (30.61)	3 (6.12)	7 (14.29)	1 (2.04)	1 (2.04)
Hypothyroidism	10 (20.41)	1 (2.04)	9 (2.04)	1 (2.04)	1 (2.04)

## DISCUSSION

The prevalence of hypothyroidism among children with nephrotic syndrome in this study was 71.01% (subclinical hypothyroidism and overt hypothyroidism). The prevalence was 41.16%, 62%, and 70.8% in a study done in Korean, Indian and Egyptian populations respectively.^1,6,8^ A case-control study from Iran found elevated TSH levels in 34.2% of nephrotic children compared to controls (10.8%).^9^

In our study, more than half of nephrotic children were of the age group between 2 to 10 years and the mean age at the time of initial diagnosis was 7.8±4.6 years. The result was comparable to the result of a study, where the mean age of the nephrotic children was 5.2±3 years and more than half of the study population was male.^9^ Our result is further supported by another study which found the mean age of nephrotic children to be 6.11±2.10 years with male: female ratio of 1.8:1.^10^

In contrast to our result of nearly equal number of hypothyroid nephrotic children below (53.06%) and above the age of 6 years (46.94%), another study found hypothyroidism more in nephrotic children below 6 years of age.^11^ The existence of a hypothyroid state in some infants with nephrotic syndrome was also shown in another study and they also recommended routine thyroid screening and early replacement therapy.^3^ A study of thyroid profile in children with untreated nephrotic syndrome had found massive urinary losses of T4, T3, Thyroid Binding Globulin (TBG), free T4 and free T3 in untreated nephrotic children. These findings provided evidence of mild hypothyroidism in children with untreated nephrotic syndrome.^12^

Elevated mean TSH (8.39±6.75 mlU/l) found in our children at the time of diagnosis of nephrotic syndrome is supported by several studies. A study from Iran also found elevated mean TSH level (11.65±6.71 mlU/L) among untreated nephrotic children.^13^ Another study carried out in Iran showed elevated TSH levels in nephrotic children compared to controls (34.2% versus 10.8%).^9^

Mean TSH (4.03±2.93) after remission of disease was lesser than mean TSH (8.39±6.75) at the time of diagnosis. However, there was not much change in mean free T3(2.65±0.62vs 2.70±0.70) and free T4 (11.58±2.74 vs 13.23±2.98) values at the time of diagnosis and after remission. But, a statistically significant result was found in a Korean study where the mean TSH level was 8.05±3.53 ng/dL at NS onset, and 4.08±2.05 ng/dL at remission.^1^ Similar to our result, a study done in Dhaka, Bangladesh also found an increase in TSH level during nephrosis (9.11 ±6.36 vs 4.2±3.6 mlU/L) and normalized during remission.^14^ A comparative study carried out in Ambala, Haryana found the mean free T3 and free T4 levels were significantly lower in cases with Nephrotic syndrome as compared to controls (Healthy children), whereas the TSH levels in cases were significantly higher than controls.^15^ ln contrast to our result, another study found that the mean serum free T4 and free T3 concentrations were significantly lower in the untreated nephrotic than in the same patients in remission, and the mean serum TSH levels were significantly higher in the untreated patients.^12^

Main limitation of the study was small sample size and inability to do 24-hour urinary T3, T4, TSH and urinary thyroid binding globulin. More studies with large sample sizes are required before making definitive conclusions on thyroid hormone replacement therapy, in addition to glucocorticoids in children with nephrotic syndrome. Hypothyroidism should be actively sought in children with nephrotic syndrome as it is a treatable complication which increases morbidity in children.

## CONCLUSIONS

The prevalence of hypothyroidism among children with nephrotic syndrome was higher than other studies done in similar settings. This study emphasizes the importance of routine thyroid function tests at the time of nephrotic syndrome diagnosis and recommends further large-scale studies to inform potential thyroid hormone replacement therapy in children alongside glucocorticoids in these children.

## References

[1] Jung SH, Lee JE, Chung WY (2019). Changes in the thyroid hormone profiles in children with nephrotic syndrome.. Korean J Pediatr..

[2] Iglesias P, Diez JJ (2009). Thyroid dysfunction and kidney disease.. Eur J Endocrinol..

[3] Mattoo TK (1994). Hypothyroidism in infants with nephrotic syndrome.. Pediatr Nephrol..

[4] Kliegman RM, Geme JWS (2019). Nelson Textbook of Pediatrics..

[5] Wassner AJ, Smith JR (2020). Nelson Textbook of Pediatrics..

[6] Basu AK, Chakraborty A, Sit S, Jana JK, Maiti S, Mandal AK (2022). Thyroid function status in nephrotic syndrome in paediatric age group: a hospital-based cross-sectional study.. Journal of Clinical and Diagnostic Research..

[7] Abd El-Aal M, Hegab AM, Masoud EE (2020). Thyroid function in children with nephrotic syndrome: a prospective hospital-based study.. Sohag Medical Journal..

[8] Sawant BU, Nadkarni GD, Thakare UR, Joseph LJ, Rajan MG (2003). Changes in lipid peroxidation and free radical scavengers in kidney of hypothyroid and hyperthyroid rats.. Indian J Exp Biol..

[9] Saffari F, Ahadi S, Dalirani R, Esfandiar N, Yazdi Z, Arad B (2020). Thyroid dysfunction in children with idiopathic nephrotic syndrome attending a paediatric hospital in Qazvin, Iran.. Sultan Qaboos Univ Med J..

[10] Vinayagam V, Uthup S, Saradakutty G, Radhakrishnan RC, Ramakrishnan L, Kailas L (2019). Thyroid function in childhood nephrotic syndrome.. Scholars Journal of Applied Medical Sciences..

[11] Choudhury J (2016). A study on thyroid function test in children with nephrotic syndrome.. Int J Contemp Pediatr..

[12] Ito S, Kano K, Ando T, Ichimura T (1994). Thyroid function in children with nephrotic syndrome.. Pediatr Nephrol..

[13] Hajizadeh N, Marashi SM, Nabavizadeh B, Elhami E, Mohammadi T, Mazloomi Nobandegani N (2015). Examine of thyroid function in pediatric nephrotic syndrome; Tehran-Iran.. Int J Pediatr..

[14] Afroz S, Khan AH, Roy DK (2011). Thyroid function in children with nephrotic syndrome.. Mymensingh Med J..

[15] Dhanjal GS, Garg M (2019). Thyroid function studies in children of nephrotic syndrome.. Int J Contemp Pediatr..

